# Fabrication and Performance Evaluation of a Cation Exchange Membrane Using Graphene Oxide/Polyethersulfone Composite Nanofibers

**DOI:** 10.3390/membranes13070633

**Published:** 2023-06-29

**Authors:** Suhun Kim, Abayomi Babatunde Alayande, Tasnim Eisa, Jaewon Jang, Yesol Kang, Euntae Yang, Moon-Hyun Hwang, In S. Kim, Kyu-Jung Chae

**Affiliations:** 1 Department of Environmental Engineering, College of Ocean Science and Engineering, Korea Maritime and Ocean University, Busan 49112, Republic of Korea; 2 Department of Marine Environmental Engineering, Gyeongsang National University, Tongyoung 53064, Republic of Korea; 3 Interdisciplinary Major of Ocean Renewable Energy Engineering, Korea Maritime and Ocean University, Busan 49112, Republic of Korea; 4 KEPCO Research Institute (KEPRI), Korea Electric Power Corporation (KEPCO), Naju 58277, Republic of Korea; 5 Starch & Sweetener R&D Department, Daesang Corporation, Seoul 07789, Republic of Korea; 6 Institute of Conversions Science, Korea University, Seoul 02841, Republic of Korea; 7 School of Earth Sciences and Environmental Engineering, Gwangju Institute of Science and Technology (GIST), Gwangju 61005, Republic of Korea

**Keywords:** electrospinning, graphene oxide, polyethersulfone, nanofiber, pore-filled, ion exchange membrane, permselectivity, areal resistance

## Abstract

Ion exchange membranes, especially cation exchange membranes (CEMs), are an important component in membrane-based energy generation and storage because of their ability to transport cations via the electrochemical potential gradient while preventing electron transport. However, developing a CEM with low areal resistance, high permselectivity, and stability remains difficult. In this study, electrospun graphene oxide/polyethersulfone (GO/PES) composite nanofibers were prepared with varying concentrations of GO. To fabricate a CEM, the pores of the electrospun GO/PES nanofiber substrates were filled with a Nafion ionomer. The pore-filled PES nanofiber loaded with 1% GO revealed a noticeable improvement in hydrophilicity, structural morphology, and mechanical properties. The 1% GO/PES pore-filled CEM was compared to a Nafion membrane of a varying thickness and without a nanofiber substrate. The CEM with a nanofiber substrate showed permselectivity of 85.75%, toughness of 111 J/m^3^, and areal resistance of 3.7 Ω cm^2^, which were 12.8%, 4.3 times, and 4.0 times better, respectively, than those of the Nafion membrane at the same thickness. The development of a reinforced concrete-like GO/PES nanofiber structure containing stretchable ionomer-enhanced membrane surfaces exhibited suitable areal resistance and reduced the thickness of the composite membrane without compromising the mechanical strength, suggesting its potential application as a cation exchange membrane in electrochemical membrane-based systems.

## 1. Introduction

The demand for green energy is increasing due to the environmental drawbacks associated with fossil fuels, such as greenhouse gas emissions [1,2]. Since the emergence of the concept of water security, the need for a safe and reliable water supply of quality and quantity has also gained steady attention [3,4,5]. Membrane-based electrochemical systems, under-researched and commercialized in water and energy applications, are the one option to solve the water and energy demands of the world. For water-based systems, efforts are underway to obtain industrial ultrapure water through electrodialysis (ED) and capacitive deionization (CDI), which utilize electrical forces to separate impurities in water, across ion exchange membranes (IEMs) [6,7,8,9]. For the energy production and storage system, one of the most active studied areas, efforts are underway regarding reverse electrodialysis (RED), which generates electricity through the movement of ions, fuel cell (FC), which converts chemical potential into electrical energy, microbial fuel cell (MFC), which reacts with micro-organisms to generate electricity, redox flow battery (RFB), which is attracting interest in the large-scale energy storage system, and electrolysis, which researches for hydrogen production [10,11,12,13,14,15,16,17]. They have a significantly lower carbon footprint than conventional methods and a high potential for stable and efficient energy generation and water production [18,19,20,21,22,23].

Electrochemical cells, IEMs, transport ions selectively based on charge and the relative size of the ions compared to the membrane. The development of novel IEMs entails incorporating high-selectivity (permselectivity), low-resistance, and anti-fouling properties while maintaining a high stability [10,24,25]. However, at a fixed thickness, there is a directly proportional relationship between resistance and permselectivity [11,12,13]. As a result, decreasing membrane thickness will reduce resistance to maintaining high permselectivity. Because thickness and mechanical strength are proportional, decreasing the thickness reduces mechanical strength and reduces the likelihood of successful application to electrochemical systems. Since membrane properties are interdependent, it is difficult to manipulate them separately. Nonetheless, increasing permselectivity and mechanical strength is critical for the broad practical application of IEMs. Therefore, researchers are investigating reducing membrane resistance through a material combination in order to form mixed-matrix membranes [14,15]. When two or more materials with dissimilar properties are combined, the benefits of each of the component materials are realized [26,27]. For example, combining polymeric materials with inorganic materials could provide benefits, such as ease of processing from the polymeric components and selectivity enhancement from the inorganic components. So, it could be possible to fabricate organic–inorganic hybrid membranes adjusted to the feasible condition. However, some combination between organic and inorganic materials means a negative interaction could occur, cross-linking could be reduced, or a non-uniform matrix could be produced due to the agglomeration of the membrane’s performance. For this reason, polyethersulfone and graphene oxide were hired as well as a known positive to composite.

Polyethersulfone (PES) is one of the most commonly used polymers for mixed-matrix membrane fabrication because of its good chemical and mechanical stability and the fact that it contains a sulfone functional group and ether linkage, but it has poor hydrophilicity [21,28,29]. To overcome this drawback, inorganic additives, such as graphene oxide (GO), can be used to improve the membrane’s properties. GO has recently garnered great attention for use in both electrochemical processes and desalination applications because of its structural and electronic properties [30,31,32,33]. GO is employed to improve the hydrophilicity, mechanical strength, and ion conductivity of membranes through its large surface area, hydrophilic nature, high electrochemical properties, and tensile modulus [34,35]. GO has become an appealing candidate for use in cation conducting systems due to its stability and variety of oxygen functional groups within its structure, which could assist in the development of hydrogen-bonded channels for cation transport [36]. Due to its interactions with the PES polymeric matrix, GO has been shown to improve the cation conductivity of polymeric IEMs [37,38].

To fabricate nanofibrous substrate, we hired the electrospinning method. Electro-spinning is a versatile technique used to prepare nanofibers that inject from the needle to collection plate in a high voltage [39,40]. The polymer solution is sprayed through a metallic needle, and the sprayed droplets are electrified and stretched and then obtained on a collecting plate in a fibrous structure due to electrostatic repulsion and coulomb forces [41,42,43]. These electrospun fibers could be fabricated uniformly with nanoscale diameters. This versatility allows the large-scale fabrication of natural, synthetic, and mixed-matrix nanofibers to take place. Electrospinning is rapidly emerging as a simple and reliable technique for producing various materials with adjustable morphologies, textures, thicknesses, mechanical properties, and porous structures from a variety of polymers [39,44]. It could be compared with other methods that fabricate nanofiber mats, such as melt-blowing, ultrasonic blowing, and self-assembly as well as a nanopore structure, such as phase inversion and stretching [45,46,47,48,49,50,51]. Compared to other methods, electrospinning can provide uniform nanoscale fibers, higher porosity, a large surface area, and a tunable structure by forming a pore structure in which the nanofibers are interconnected [52,53]. Also, these can be produced in a simple set-up, at a low cost, and whilst reducing waste materials or water [41,48].

Based on the electrospun nanofiber substrate, pore-filling was prepared to fabricate IEM. Pore-filling is one of the techniques used to fabricate IEM by filling nanometric size pores with conductive polymers such as Nafion, Aciplex, and Flemion [54,55,56]. The polymeric electrolyte can have an affinity for the pore-filled substrate with a high flexibility and even distribution with a uniform structure. In this study, Nafion ionomer, one of the most attractive commercialized materials for the electrochemical system, was employed for polymeric electrolyte solution [54,55,57]. This pre-fluorinated sulfonic acid polymer-based electrolyte has a high ion conductivity and good mechanical and chemical stability. So, the Nafion ionomer fully filled the void of the substrate, among electrospun nanofibers, forming a reinforced concrete-like structure.

In this study, our hypothesis is that the incorporation of GO into PES will enhance the mechanical strength and hydrophilicity of the resulting membrane. Additionally, we propose that pore-filling the membrane with Nafion ionomer will enhance its performance by creating a fully filled void structure, thereby improving ion transport and over-all membrane properties. The high mechanical strength provided by the incorporation of GO will allow a reduction in membrane thickness to occur without compromising its strength, leading to a further reduction in membrane resistance.

## 2. Materials and Methods

### 2.1. Materials

Polyethersulfone (BASF Co., Germany) was used after drying at 120 °C for at least 24 h. Graphite power (325 mesh) was purchased from Alfa Aesar, USA. Nafion solution (D2021, dispersion alcohol-based 1100 EW at 20 wt%, Fuel Cell Store, USA) was employed as the pore-filling ionomer. Deionized water (DI) was obtained from Milli-Q System (Millipore, USA). Dimethylacetamide (DMAc), hydrochloric acid (HCl, mass fraction of 35%), sulfuric acid (H_2_SO_4_, mass fraction of 98%), potassium permanganate (KMnO_4_), hydrogen peroxide (H_2_O_2_, mass fraction of 30%), sodium chloride (NaCl), and sodium hydroxide (NaOH) were provided by Daejung Chemical, Republic of Korea. All chemicals were used without further purification.

### 2.2. Nanofiber Preparation

**Figure 1:**
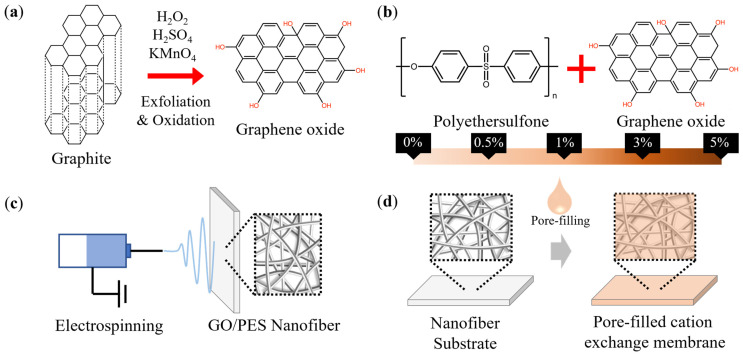
The process of membrane fabrication: (**a**) exfoliation and oxidation of graphite to graphene oxide, (**b**) preparation of a GO and PES mixture, (**c**) electrospinning, and (**d**) pore-filling.

The GO was prepared using a modified Hummers method, as described in [58,59,60]. Briefly, graphite powder was thoroughly mixed with H_2_SO_4_. The mixture was placed in an ice bath to neutralize the temperature of the exothermic reaction, after which KMnO4 was added and stirred for at least an hour until it reached a homogenous state. The solution was placed in a 35 °C water bath and stirred for 24 h. Next, H_2_O_2_ was dripped into the solution. During this process, the mixture’s color gradually changed from yellow to brown, and gas was produced as a result of oxidation. Following oxidation, the solution was centrifuged and repeatedly washed with 10% HCl solution and DI water. After washing, the solution was dialyzed for 24 h to remove impurities before being dried at 60 °C to produce dark brown GO. Figure 1a provides a concise and straight forward illustration of the concept.

The polymer mixtures used for electrospinning were prepared by mixing the PES solution with different concentrations of the obtained GO (0, 0.5, 1, 3, 5%) to obtain five types of nanofibers (N-0, N-0.5, N-1, N-3, N-3, respectively). The mixture solutions were prepared solute/solvent *w*/*v*% as the 25 *w*/*v*%, and the percentage of GO was calculated as the weight ratio between GO. The brief scheme of the GO/PES mixture and fabrication of the nanofiber are explained in Figure 1b,c. The spinning conditions were a temperature of 20 ± 2 °C, a humidity of 50% ± 5%, and a spinning time of 40 min. Nanofibers were collected on a drum with an aluminum plate that rotated at 200 rpm. A lab-scale electrospinning setup (NanoNC Co., Ltd., Republic of Korea) was used to fabricate the nanofibers.

### 2.3. Pore-Filling

The pore-filling of the nanofiber is simply shown as Figure 1d. The Nafion ionomer was used for pore-filling after dilution with ethanol at a 1:3 *v*/*v* ratio. The nanofiber mat was placed in a Petri dish, and the prepared Nafion solution was poured slowly and evenly over the top. The membrane was heat treated on a hot plate at 50 °C for 12 h and then at 80 °C for one hour. The pore-filled membrane was named “pore-filled cation exchange membrane (PFM)” for this study. Three kinds of nanofiber-free cast Nafion membranes (CNM-1, CNM-2, CNM-3) were fabricated to compare the relationship between thickness and pore-filling. CNM-1 was fabricated with the same amount of Nafion solution that was used to prepare PFM. CNM-2 and CNM-3 were prepared with an additional 5 mL and 10 mL of Nafion solution compared to CNM-1, respectively. Except for the amount of Nafion solution used, the entire fabrication process was identical to that of PFM. 

### 2.4. Characterization

The morphology of the GO material and membranes was observed using a field emission scanning electron microscope (FE-SEM, Hitachi, S-4700, Japan), and the fiber diameter was calculated using image software (https://imagej.nih.gov/ij/download.html, accessed on 25 April 2023). The water contact angle (WCA) was measured with a contact goniometer (Phoenix 300, Surface Electro Optics Co., Ltd., Republic of Korea), which dropped a single water droplet onto the dried samples and took images in five seconds. Toughness was analyzed to determine the mechanical strength of the material. The definition of toughness is the ability to withstand elastic deformation, a measure of the amount of energy a material can absorb before actual cracking or fracture occurs. Based on this definition, a universal testing machine (Instron 5567, USA) was used to simultaneously measure the tensile strength and strain to obtain a graph, which was then integrated to determine the amount of energy required to fracture the membrane. The thermogravimetric analyzer (TGA, TGA8000, Perkin Elmer, USA) was measured for thermal stability until 700 °C by 20 °C/min in N_2_ atmosphere. The composition of the nanofibers was analyzed using an X-ray diffraction (XRD) spectroscope (D/Max-2500, Rigaku, Japan) and Raman spectroscopy (LabRAM HR Evolution, Horiba, France). A zeta potential analyzer (ELSZ-2000, Otsuka Electronics Corp., Japan) was also used to measure the zeta potential of the membrane surface.

**Figure 2:**
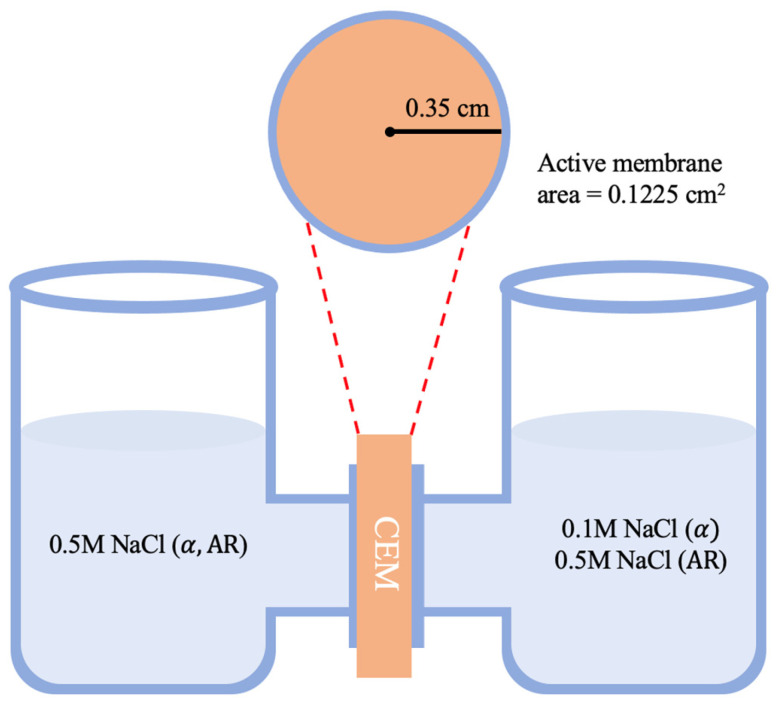
Schematic diagram of two-compartment cell experiments for permselectivity and areal resistance.

A two-compartment cell experiment with direct current was used to measure permselectivity (α). The electrolyte solutions were prepared with 0.1 M and 0.5 M sodium chloride concentrations, and a membrane was placed between the two compartments as described in Figure 2. The solutions were continuously stirred, and voltage (∆V_measured_) was measured with a multimeter and an Ag/AgCl electrode. As a result, permselectivity was calculated using Equation (1), where F is the Faraday constant, R is the gas constant, T is the absolute temperature, z is the valence of the ions, and C_H_ and C_L_ are the high and low salinity concentrations, respectively.

(1) $$\alpha \left(\%\right)=\frac{\Delta V_{\mathrm{measured}}}{\Delta V_{\mathrm{theoretical}}}\times 100$$

(2) $$\Delta V_{\mathrm{theoretical}}=\frac{\mathrm{RT}}{\mathrm{zF}}\;\mathrm{In}\left(\frac{C_{H}}{C_{L}}\right)$$

The two-compartment cell was also used to estimate the areal resistance as described in Figure 2. The electrolyte was 0.5 M NaCl solution, and the active membrane area was 0.1225 cm^2^. The calculation was performed using Equation (4).

(3) $$R_{\mathrm{membane}}\left(\Omega \right)=R_{\mathrm{cell}}-R_{\mathrm{electrolyte}}$$

(4) $$\mathrm{Areal}\;\mathrm{resistance}\left({\Omega \;\mathrm{cm}}^{2}\right)=R_{\mathrm{membrane}}\times \mathrm{Active}\;\mathrm{membrane}\;\mathrm{area}$$

The weight difference between dry and wet membranes was used to calculate the water uptake (WU). The wet membrane was dried for at least 24 h at 80 °C in a dry oven then soaked in water for 24 h to ensure the complete absorption of water molecules into the membrane. Any excess water on the membrane’s surface was removed. The WU was calculated using Equation (5), where w_wet_ and w_dry_ represent the membrane weight in wet and dry states, respectively.

(5) $$\mathrm{Water}\;\mathrm{Upate}\left(\mathrm{wt}\%\right)=\frac{w_{\mathrm{wet}}-w_{\mathrm{dry}}}{w_{\mathrm{wet}}}\times 100$$

Titrations with NaCl and NaOH solutions were used to calculate the ion exchange capacity (IEC). After immersing the membrane in a 2 M NaCl solution for 24 h, the solution containing the membrane was titrated with a 0.1 M NaOH solution until a pH of 7 was reached. Therefore, the calculation was performed as shown in Equation (6), where C_NaOH_ and V_NaOH_ are the concentration and volume of the titrated NaOH solution, respectively, and m_dry_ is the weight of the dry membrane.

(6) $$\mathrm{Ion}\;\mathrm{Exchange}\;\mathrm{Capacity}\left(\mathrm{meq}/g_{\mathrm{dry}}\right)=\frac{C_{\mathrm{NaOH}}\times V_{\mathrm{NaOH}}}{m_{\mathrm{dry}}}$$

## 3. Results

### 3.1. Effect of GO on the Electrospun PES Nanofiber

**Figure 3:**
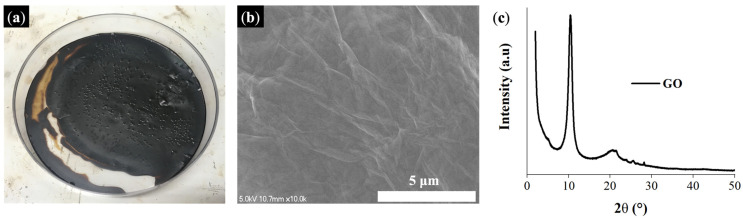
Prepared GO: (**a**) digital picture of GO, (**b**) SEM image of GO, and (**c**) XRD analysis of GO.

Figure 3 depicts the digital image, SEM image, and XRD data of the GO prepared using a modified Hummers method. The SEM image revealed a wrinkled and multi-layered surface for the GO nanosheets. Furthermore, XRD data indicated that the prepared GO had a significant peak near 2θ = 10° [61]. Evidently, as-prepared GO was exfoliated from graphite and successfully oxidized.

**Figure 4:**
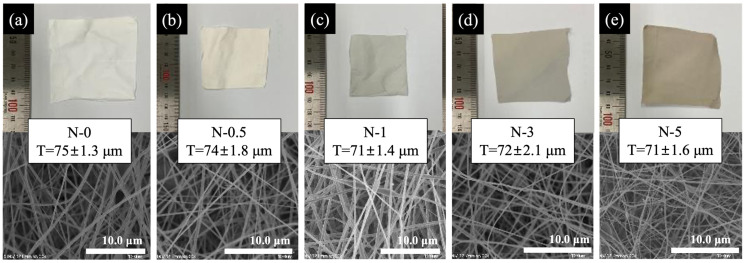
Digital picture and SEM image; thickness profiles of electrospun nanofibers: (**a**) N-0, (**b**) N-0.5, (**c**) N-1, (**d**) N-3, (**e**) N-5.

Figure 4 shows the digital pictures, SEM images, and thickness profiles of electrospun nanofibers at various GO concentrations. The brown color of the GO in nanofiber membranes became more pronounced (i.e., from white to brown) as the concentration of GO increased from left to right in the digital images. As shown in the SEM images of each nanofiber and its diameter distribution, there was no evidence of a significant change in the nanofiber diameter and structure due to a change in the GO concentration, implying that adding GO had no effect on the fiber’s condition.

**Figure 5:**
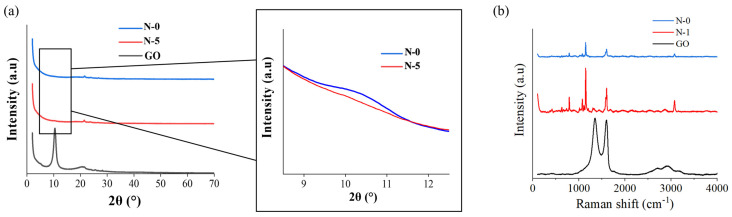
XRD and Raman spectroscopy comparison among GO and nanofibers: (**a**) XRD data of N-0, N-5, GO; (**b**) Raman data of N-0, N-1, GO.

The thickness of the nanofiber mats was consistent because the same volume of solution was injected for the same amount of time. Figure 5 depicts an XRD and Raman spectroscopy analysis of nanofibers to confirm the status of composition between GO and PES. In the XRD analysis, the peak of N-5 containing 5% GO was similar to the peak of GO at 2θ = 10°. In the Raman spectroscopy, N-1 showed a higher intensity near 1500 and 2800 cm^−1^ where the GO peaks compared to N-0. The results imply that GO and PES were successfully combined. 

When GO was added to PES, the properties changed. As shown in Table 1, the WCA of the nanofibers with GO decreased by approximately 10° compared to that of the pure PES nanofiber, reaching a minimum of 63.41° at N-5. Therefore, regardless of the concentration, the hydrophilicity of the nanofiber membrane was improved when GO was present. As also shown in Table 1, adding GO improved the toughness over pristine PES nanofibers. Among the nanofibers, N-1 with 1% GO demonstrated the highest mechanical strength. It was deduced that when GO and PES were combined, a strong bond was formed between the functional groups of the materials, increasing the strength of the membrane [59,60]. 

**Figure 6:**
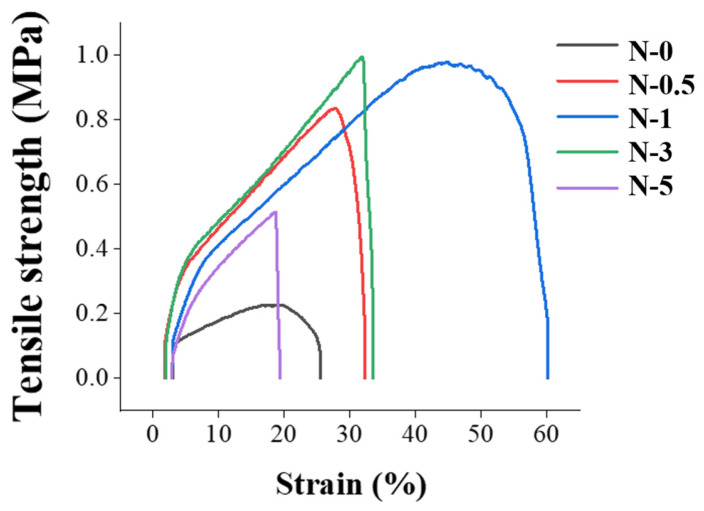
Tensile strength–strain curves of nanofibers.

Furthermore, the electrochemical properties of GO may aid in drawing the electrospinning jet more evenly and uniformly than with pristine PES, thereby improving the mechanical strength of the nanofiber [43,62]. However, when the amount of GO exceeded the binding stoichiometry between GO and PES, the mechanical strength of the membrane decreased, as shown in Table 1 and Figure 6, with N-3 and N-5 being weaker than N-1. Moreover, when the nanofibers were spun, excess GO coagulated, caused by a higher concentration of GO. The coagulated GO regions acted as a defect, reducing the mechanical strength. Since tensile strength and strain have the same tendency as toughness, it was concluded that N-1 had the best mechanical strength.

### 3.2. Effects of the Pore-Filled Structure Design on Membrane Performance

**Figure 7:**
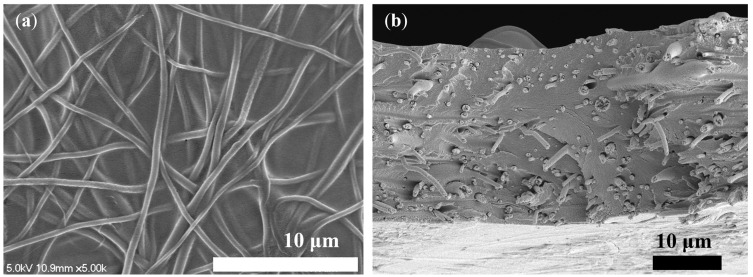
SEM image of the pore-filled membrane. (**a**) top view; (**b**) cross-sectional view.

Following the comparison of nanofibers, N-1 was chosen as the pore-filling substrate. Therefore, PFM was fabricated using the smooth and polymeric Nafion ionomer solution as the pore-filling electrolyte, which filled the pores of the N-1 nanofiber, as shown in the SEM image in Figure 7. The Nafion ionomer fully filled the void of the substrate, among electrospun nanofibers, forming a reinforced concrete-like structure. It was confirmed that there were no voids inside the PFM through SEM images. Thus, the pore-filling procedure was completed successfully.

**Figure 8:**
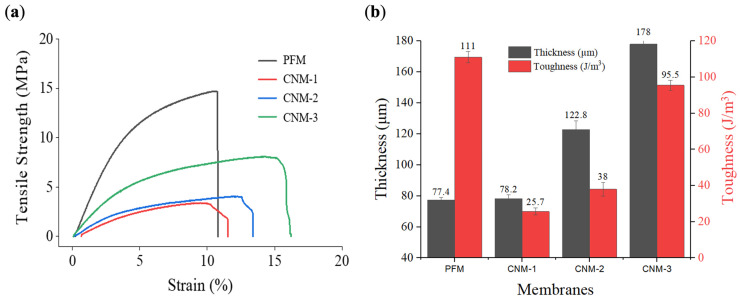
Mechanical stability of membranes: (**a**) tensile strength–strain curves of membranes; (**b**) relationship between thickness and toughness of membranes.

Figure 8a shows the tensile strength–strain curves of PFM, CNM-1, CNM-2, and CNM-3. PFM and CNM-1 had the same thickness because the same volume of pore-filling electrolyte was used. As the volume of the pore-filling solution increased, the thickness of CNM-2 and CNM-3 increased. Correspondingly, membranes could be compared in terms of the mechanical properties, such as toughness, in relation to thickness differences. The curves were increased by the thicker casted Nafion membrane, respectively. The toughness of cast Nafion membranes without a nanofiber mat was proportional to thickness. Casted Nafion membranes showed a higher strain than PFM; however, they were not enduring the tensile. So, PFM, the thinnest membrane with a nanofiber substrate, had the highest toughness overall. The structure improved via pore-filling with Nafion ionomer into the nanofiber with a higher mechanical stability. This is analogous to the formation of a reinforced concrete structure [63,64]. Therefore, improving the membrane structure may result in increased toughness despite the lower thickness.

**Figure 9:**
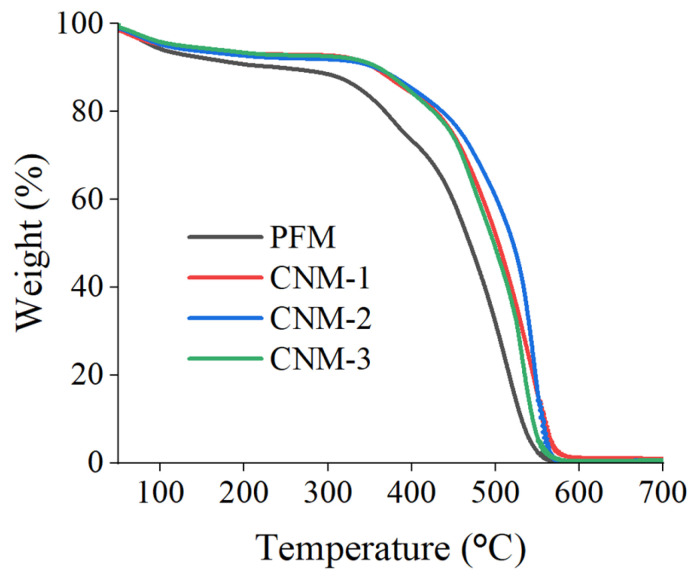
TGA curves of membranes.

Figure 9 shows the TGA curves of the membranes. The casted Nafion membranes show that decreases in similar curves started near 400 °C and dramatically dropped near 500 °C. However, PFM, containing the GO/PES composite nanofiber, loses weight faster than casted Nafion membranes. GO does not perform in a way which ensures a high-temperature membrane, as the references reported [65,66]. PES has a higher thermal stability than other materials (GO, Nafion); however, its weight ratio of PES in the membrane, mostly composed by Nafion, is not enough to induce an increase in the thermal stability [67,68].

Table 2 shows the physical and electrochemical properties of the membranes. PFM had a lower surface charge than the CNMs because of the GO which was added to the nanofiber. PES had a surface charge of approximately −30 mV and GO had a surface charge of approximately −50 mV at a pH of 7 [69,70]. Therefore, incorporating GO into the nanofiber resulted in a difference in the zeta potential. Higher WU and permselectivity could be caused by the negative surface charge. In the case of the CNMs, the WU was proportional to membrane thickness, implying that the thicker the membrane, the more water molecules which can be contained. In the case of PFM, WU was improved by 12% when compared to CNM-1 at the same thickness. This result was caused by the negative surface charge of PFM, which attracted water molecules and thus increased hydrophilicity [60,71]. The lower WCA of PFM compared to the CNMs demonstrated increased hydrophilicity. The permselectivity of PFM was 13% higher than that of CNM-1 and slightly higher than CNM-2. The lower zeta potential, which could effectively reject co-ions while allowing counter-ions, explained the high permselectivity at the same thickness. The higher hydrophilicity and WU of PFM, in accordance with the general ion transfer mechanism inside the membranes, can induce more effective ion transport. 

According to the IEC, the number of ions that can be contained by a thicker membrane is proportional to its thickness. PFM had an IEC of 0.88 meq/g_dry_, which is 3.3% lower than CNM-1′s IEC of 0.91 meq/g_dry_. The structure of PFM could hinder ion exchange due to the presence of a nanofiber substrate inside the membrane. This substrate may act as a barrier and create a lack of electrolyte-filled pores, which could impede the movement of ions across the membrane. PFM had up to four times the areal resistance of CNM-1, the thinnest membrane, and six times the areal resistance of CNM-3, the thickest membrane. This may be explained similarly to permselectivity. The area resistance is proportional to the thickness; as the thickness increases, the ion passage decreases, and the area resistance increases. The high WU of PFM allowed effective ion transport to take place, and the negative surface charge promoted counter-ion penetration through the membrane [13]. Therefore, PFM, the thinnest cation exchange membrane, outperforms the thickness-controlled Nafion membranes.

## 4. Conclusions

In this study, the cation exchange membrane was fabricated by the sequence of electrospinning for nanofiber substrate and pore-filling. An electrospun nanofiber was selected from five different concentrations of GO based on the PES polymer. The addition of GO increased the electrochemical and mechanical properties of the PES nanofiber due to the more negatively charged surface that induced a hydrophilic atmosphere and bonding force between the polymer molecules. Furthermore, by combining PES and 1% GO, the nanofiber achieved a toughness of up to 39.9 J/m^3^. Based on this substrate, for pore-filling followed by the Nafion ionomer, we fabricated a pore-filled cation exchange membrane with electrolyte solution which fully filled the void of the substrate. This was compared with three different recast Nafion membranes in order to demonstrate it structurally modified formation, high strength, high permselectivity, high hydrophilicity, and low areal resistance. The mechanical strength was greatly improved up to 111.4 J/m^3^ compared to that of the nanofiber mat alone and the cast Nafion membrane. The strength could be further improved by combining high-tensile-strength nanofibers with stretchable Nafion electrolyte. This structural engineering resulted in an IEM with a reinforced concrete-like structure. Even as its thickness decreased, the IEM exhibited improved mechanical strength and resistance due to the smoother flow of ions through ion channel structures provided by the nanofibers. Furthermore, because GO increased the membrane’s zeta potential, a more negatively charged membrane surface effectively selected ions to pass or reject, increasing permselectivity up to 85.75% and decreasing areal resistance up to 3.7 Ω cm^2^. In conclusion, the membrane designed in this paper improved mechanical strength, permselectivity, and areal resistance through material combination via electrospinning and structure modification with pore-filling and has a promising future applied to system adaptation.

## Figures and Tables

**Table 1:** Properties of nanofibers.

Nanofiber	Fiber Diameter (nm)	Porosity (%)	Pore Size (µm)	Thickness (µm)	Toughness (J/m^3^)	WCA (°)
N-0	359	62.56	383.24 + 17.94	75.3	4.12	77.09
N-0.5	306	64.10	347.52 + 24.48	74.8	17.51	67.72
N-1	368	69.02	345.73 + 14.91	71.4	39.90	66.05
N-3	365	66.51	372.1 + 28.14	72.1	20.04	63.60
N-5	298	64.62	35.6 + 15.04	71.6	5.62	63.41

**Table 2:** Physical and electrochemical properties of the membranes.

Membrane	Thickness (μm)	Zeta Potential (mV)	WCA (°)	WU (%)	Permselectivity (%)	IEC (meq/g_dry_)	AR (Ω cm^2^)
PFM	77.4	−34.4	62.2	22.7	85.75	0.85	3.7
CNM-1	78.2	−31.7	68.2	20.3	75.99	0.91	14.7
CNM-2	122.8	−32.1	67.2	30.3	84.7	0.93	16.5
CNM-3	178.0	−31.4	69.2	33.9	92.88	0.96	20.1

## Data Availability

Not applicable.
